# Supplementary material for: The value of nanocarbon contrast methylene blue based on dye-based tracer technology in sentinel lymph node biopsy for breast cancer: a systematic review and meta-analysis
Source: PeerJ. 2025 Jun 11;13:e19546. doi: 10.7717/peerj.19546 (PMC12166851; doi:10.7717/peerj.19546)
Supplement: Supplemental Information 1 [file peerj-13-19546-s001.docx]

|  |  |  | **NCS-group** | | | | | **MBI-group** | | | | | |
| --- | --- | --- | --- | --- | --- | --- | --- | --- | --- | --- | --- | --- | --- |
| **Author** | **Age** | **Year** | **TP** | **FP** | **FN** | **TN** | **Total** | **TP** | **FP** | **FN** | **TN** | **Total** | |
| **Jie GE** | **28-73** | **2011** | **21** | **0** | **2** | **41** | **64** | **13** | **6** | **2** | **20** | **51** | |
| **Lijie YANG** | **Unclear** | **2011** | **11** | **2** | **1** | **28** | **42** | **12** | **3** | **1** | **28** | **44** | |
| **Yi ZHOU** | **28-76** | **2012** | **29** | **4** | **2** | **43** | **78** | **23** | **11** | **1** | **30** | **65** | |
| **Qimou LIN** | **26-72** | **2012** | **25** | **2** | **1** | **36** | **64** | **26** | **8** | **1** | **22** | **57** | |
| **Xuan SUN** | **28-68** | **2013** | **37** | **0** | **2** | **49** | **88** | **30** | **0** | **4** | **56** | **90** | |
| **Xiufeng Wu** | **24-72** | **2015** | **24** | **0** | **3** | **56** | **83** | **16** | **0** | **3** | **54** | **73** | |
| **Pangzhou CHEN** | **33-48** | **2015** | **9** | **2** | **0** | **39** | **50** | **6** | **0** | **3** | **27** | **36** | |
| **Fei MAI** | **27-69** | **2015** | **19** | **0** | **2** | **22** | **43** | **14** | **0** | **1** | **16** | **31** | |
| **Lei WANG** | **22-68** | **2017** | **12** | **1** | **1** | **39** | **53** | **15** | **6** | **1** | **25** | **47** | |
| **Qin ZHU** | **30-65** | **2017** | **50** | **3** | **3** | **90** | **146** | **51** | **7** | **5** | **85** | **148** | |
| **Jingwen ZHANG** | **average50.5** | **2017** | **20** | **0** | **2** | **118** | **140** | **31** | **0** | **6** | **108** | **145** | |
| **Dihang LI** | **24-71** | **2018** | **15** | **0** | **1** | **31** | **47** | **14** | **0** | **1** | **28** | **43** | |
| **Xiao QI** | **30-84** | **2018** | **16** | **0** | **1** | **35** | **52** | **17** | **0** | **2** | **31** | **50** | |
| **Peiyang WU** | **29-72** | **2019** | **15** | **0** | **1** | **30** | **46** | **11** | **0** | **2** | **17** | **30** | |
| **Xiaomin LIU** | **32-71** | **2019** | **15** | **1** | **2** | **41** | **59** | **14** | **1** | **3** | **41** | **59** | |
| **Guangfa XIA** | **25-82** | **2019** | **25** | **1** | **1** | **59** | **86** | **11** | **1** | **1** | **64** | **77** | |
| **Ningxiang JIANG** | **36-88** | **2021** | **8** | **1** | **1** | **20** | **30** | **6** | **3** | **2** | **19** | **30** | |
|  |  |  |  |  |  |  | **1171** |  |  |  |  |  | **1076** |
